# Porphyrin-Based MOF Thin Film on Transparent Conducting Oxide: Investigation of Growth, Porosity and Photoelectrochemical Properties

**DOI:** 10.3390/molecules28155876

**Published:** 2023-08-04

**Authors:** Ben Gikonyo, Fangbing Liu, Saly Hawila, Aude Demessence, Herme G. Baldovi, Sergio Navalón, Catherine Marichy, Alexandra Fateeva

**Affiliations:** 1 Laboratoire des Multimatériaux et Interfaces, Université Lyon, Université Claude Bernard Lyon 1, UMR CNRS 5615, F-69622 Villeurbanne, France; 2 Université Lyon, Université Claude Bernard Lyon 1, Institut de Recherches sur la Catalyse et l’Environnement de Lyon (IRCELYON), UMR CNRS 5256, F-69626 Villeurbanne, Franceaude.demessence@ircelyon.univ-lyon1.fr (A.D.); 3 Departamento de Química, Universitat Politècnica de València, C/Camino de Vera, s/n, 46022 Valencia, Spain; hergarba@cam.upv.es (H.G.B.); sernaol@doctor.upv.es (S.N.)

**Keywords:** metal organic framework, thin films, porosity, transparency, optical properties, absorption, luminescence, atomic layer deposition, solvothermal conversion

## Abstract

Synthesizing metal-organic frameworks (MOFs) composites with a controlled morphology is an important requirement to access materials of desired patterning and composition. Since the last decade, MOF growth from sacrificial metal oxide layer is increasingly developed as it represents an efficient pathway to functionalize a large number of substrates. In this study, porphyrin-based Al-PMOF thin films were grown on conductive transparent oxide substrates from sacrificial layers of ALD-deposited alumina oxide. The control of the solvent composition and the number of atomic layer deposition (ALD) cycles allow us to tune the crystallinity, morphology and thickness of the produced thin films. Photophysical studies evidence that Al-PMOF thin films present light absorption and emission properties governed by the porphyrinic linker, without any quenching upon increasing the film thickness. Al-PMOF thin films obtained through this methodology present a remarkably high optical quality both in terms of transparency and coverage. The porosity of the samples is demonstrated by ellipsometry and used for Zn(II) insertion inside the MOF thin film. The multifunctional transparent, porous and luminescent thin film grown on fluorine-doped tin oxide (FTO) is used as an electrode capable of photoinduced charge separation upon simulated sunlight irradiation.

## 1. Introduction

Beyond the classical routes of metal-organic framework (MOF) synthesis, their growth from sacrificial metal oxides is being increasingly investigated due to the versatility and the benefits offered by the patterning and templating effect of this approach. Indeed, metal oxides are less soluble inorganic precursors for MOFs compared to the metal salts; therefore, their use allows performing the synthesis while withholding the morphology of the parent oxide. This methodology was introduced in 2012 by Kitagawa et al. as the coordination replication method to process MOFs from the sol-gel elaborated alumina architectures by reacting them in a ligand-containing solution [1]. The method was then extended to the use of metal oxide deposited in the vapor phase by the means of atomic layer deposition (ALD), enabling conformality and thickness to be atomically controlled [2]. Later, it was further extended through the ligand reactions in the vapor phase by chemical vapor deposition (CVD) conversion to reach MOF [3]. Since then, conversion of ALD-processed metal oxide became appealing for the elaboration of MOF coatings on materials of chosen nature, shape and geometry [4,5,6,7,8], although it remains less explored for the growth of 2D film architectures [9,10,11]. Yet, regarding MOF thin films elaboration, this approach allows us to address the major challenges in the field, such as the film thickness control, optical quality, grain size homogeneity, coverage and strong interaction (adherence) between the MOF and the surface.

**Figure 1:**
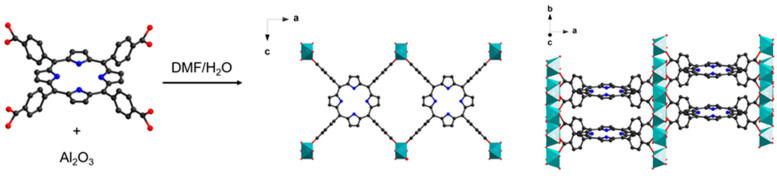
Synthesis of Al-PMOF from ALD-deposited alumina; the structure of Al-PMOF is shown along two different directions.

Given our major interest in the development of porphyrin-based MOFs as functional materials for applications in photocatalysis, electrocatalysis and sensing, we aim to develop a strategy to process porphyrin-based MOFs into thin films of high optical quality, which is necessary to improve the efficiency of light-triggered processes. For this purpose, transparent conducting fluorine-doped tin oxide (FTO) was chosen as substrate due to its stability, optical and electrical properties [12], and the chemically robust and intrinsically porous porphyrin-based Al-PMOF [13] thin film growth was investigated. Al-PMOF is made up from the coordination of the tetracarboxyphenyl porphyrin (TCPP) with trivalent aluminum ions. The inorganic building unit is composed of chains of corner-sharing Al octahedra (connected by hydroxy bridges), and each organic porphyrin ligand is connecting eight aluminum ions from four different chains (Figure 1). The material presents pore channels running in the three directions with a BET surface area of 1400 m^2^·g^−1^ and a thermal stability under air up to 350 °C. The remarkable chemical stability of Al-PMOF in water and in acidic conditions allowed its use in the powder form for major catalytic transformations such as photocatalytic hydrogen evolution [13], electrocatalytic CO_2_ [14], O_2_ [15] and N_2_ [16] reductions. Noticeably, when built from the free base porphyrin, Al-PMOF can be further tuned by post-synthesis metal insertion inside the porphyrinic core to modify the functionality [13,15,17]. 

Al-PMOF growth from ALD-deposited metal oxide was recently implemented for the fabrication of protective fabrics [6], electrodes [11] and mesoscopic constructs [18]; however, no thorough study of the film growth process, film morphology and its optical properties is reported to date. In our goal to reach light-responsive MOF architectures, we investigate the growth of Al-PMOF thin films from ALD-coated FTO substrates, characterize their crystallinity and morphology as well as their optical and electrochemical properties. 

## 2. Results and Discussion

### 2.1. Solvent Effect on the Conversion of Alumina to Al-PMOF

**Figure 2:**
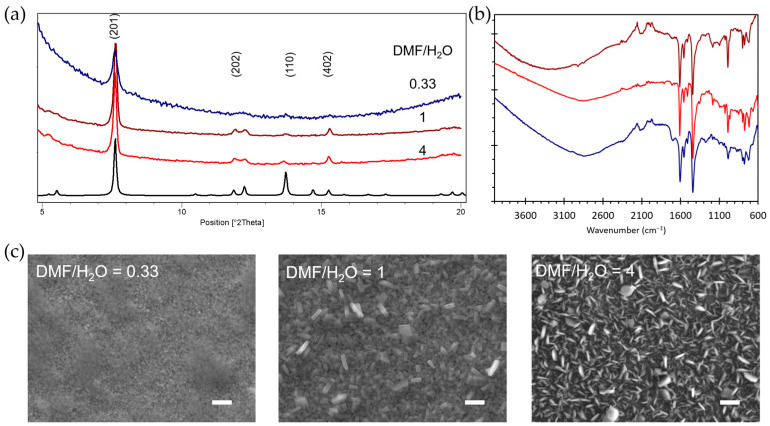
(**a**) PXRD patterns of Al-PMOF grown from alumina on FTO (grown with 300 ALD cycles) with variable solvent composition and the calculated pattern in black, (**b**) FT-IR spectra of the Al-PMOF thin films obtained with variable solvent composition (same color code as in (**a**)), (**c**) SEM images of the Al-PMOF thin films obatined with various solvent compositions; the scale bar represents 1 micrometre.

The liquid-phase conversion of the metal oxide to MOF proceeds through a dissolution–crystallization process at the interface between the metal oxide and the ligand-containing solution. An interplay between the metal oxide dissolution and the MOF formation rates is of major importance to reach MOF growth on the solid surface rather than homogeneous crystallization in solution from the dissolved metal oxide species. As previously reported, the kinetics of both metal oxide dissolution and coordination reaction are greatly impacted by the reaction experimental parameters such as the solvent composition and the reaction temperature [19]. Regarding the latter, Al-PMOF crystallization is promoted by high temperatures (the bulk sample synthesis is performed in water at 180 °C) [13] that are compatible with the use of the robust FTO substrate. Based on this and the previous studies, the reaction temperature was set here at 150 °C (see Section 3 for experimental details). It was previously reported that dimethylformamide (DMF)/water mixtures are well suited to balance between the rapid dissolution of alumina in the aqueous phase and the porphyrin solubility in the organic phase. Building on the reported conditions [5,14,18], we investigated the conversion reaction for three different solvent compositions with DMF/water ratios of 1/3, 1 and 4. In each case, aluminum coordination by the porphyrinic ligand was evidenced by the FT-IR spectroscopy with the clear signals of asymmetric and symmetric carboxylate vibrations at 1610 cm^−1^ and 1440 cm^−1^, respectively (Figure 2b). Nonetheless, powder X-ray diffraction (PRXD) and scanning electron microscopy (SEM) data indicated major differences. At low DMF content, the morphology of the MOF crystallites is ill-defined (Figure 2c) and the PXRD patterns display weak intensity peaks (Figure 2a). This can be attributed to the rapid hydrolysis of alumina in the water-rich solvent leading to solution release with homogeneous nucleation and poor MOF surface coverage. When the DMF/water ratio is raised to 1, well-defined prismatic MOF crystallites are evidenced by SEM, and a further increase in DMF content leads to a shape change of the crystallites toward a tabular habit (Figure 2c). This morphology change may be explained by the slower alumina dissolution and diffusion near each nucleation site in DMF-rich solvent, resulting in thinner crystals. Interestingly, some degree of orientation is noticeable from the SEM images, as most of crystallites grows with the largest facets perpendicular to the substrate (Figure 2c). The PXRD patterns show a substantial increase in crystallinity for the DMF/water ratios of 1 and 4 (Figure 2a). When compared to the bulk Al-PMOF powder diffraction pattern, the relative intensities of the (2,0,1), (2,0,2) and (4,0,2) diffraction peaks are significantly enhanced, which suggests some degree of preferential orientation during the film growth process, leading to a majority of crystallites positioned with the b axis running parallel to the substrate. From this set of experiments, a DMF/water ratio of 1, limiting the amount of toxic DMF solvent, was chosen to explore further Al-PMOF growth and the properties of the composite thin film. 

### 2.2. Impact of the Alumina Thickness on Al-PMOF Morphology

**Figure 3:**
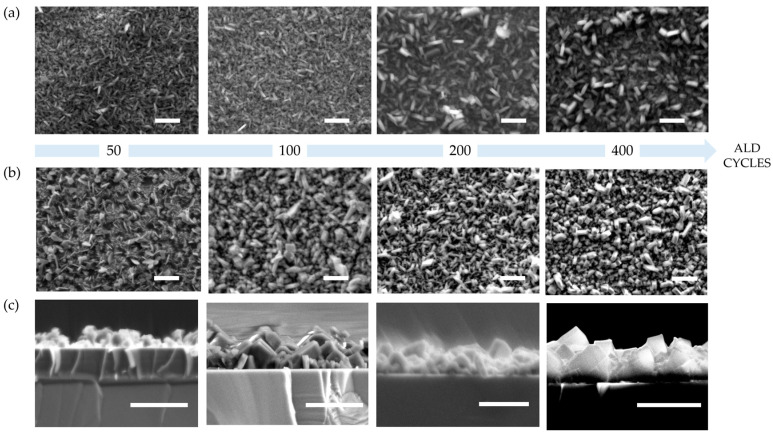
SEM images of Al-PMOF grown from alumina deposited with variable number of cycles, samples grown on FTO substrates (**a**), samples grown on Si substrates (**b**), cross-section of samples grown on Si substrates (**c**), scale bar 1 micrometer.

ALD is particularly well suited to grow films of a controlled thickness. Spectroscopic ellipsometry allows us to determine that alumina is deposited with a growth per cycle (GPC) of ca. 0.07–0.1 nm. Alumina films with variable thicknesses were grown on silicon wafers and FTO through 50, 100, 200 and 400 ALD cycles. Silicon wafers were used along with the FTO substrates to allow easy cross-sectional microscopy, assuming that the thin film growth would proceed similarly on both alumina-coated substrates. The impact of the parent alumina thickness on the morphology, crystallites size and thickness of MOF film was first studied by SEM. Figure 3 shows that the MOF crystallites on FTO and Si substrates follow a gradual size increase while increasing the thickness of the parent alumina layer. Crystallites grown from the thinnest (ca. 5 nm) alumina layer are on average 300 nm long on both substrates. When the alumina layer is increased to about 30–40 nm, MOF crystallites size increases to approximately 500 nm long on both substrates. Along with the size, the morphology change is noticeable especially on Si substrate: from tabular to prismatic with the increasing alumina thickness.

**Figure 4:**
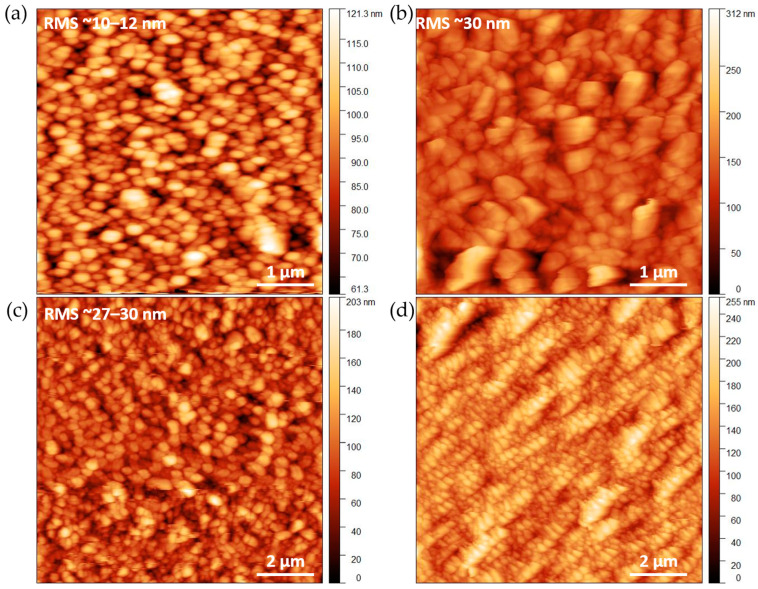
AFM images of bare FTO covered glass slide (**a**), and AlPMOF films grown on FTO from alumina layers deposited with 100 (**b**,**d**) and 200 (**c**) ALD cycles, respectively.

Atomic force microscopy (AFM) imaging confirms the grain evolution observed by SEM. Figure 4c,d shows the increase in the grain size as a function of the film thickness. Well-faceted grains, in agreement with prismatic shape, are observed (Figure 4b and Figure S1). Remarkably, increasing the layer thickness has little effect on the roughness (Figure 4b,c and Figure S1). Regardless, the substrate and the starting thickness of the alumina layer, as well as the obtained Al-PMOF films present an extracted root mean square (RMS) roughness of 30 nm on FTO (bare FTO RMS = 10 nm) and up to 55 nm for the thickest sample grown on silicon (bare Si RMS = 0.3 nm, Figure S1). 

Thin films grown on Si wafer were cleaved to perform cross-sectional SEM analysis. The corresponding thickness measurements show a gradual increase from 170 to 300, 420 and 650 nm average thickness for the samples obtained from 50, 100, 200 and 400 ALD cycles, respectively (Figure 3c). The plot of the Al-PMOF thickness against the starting alumina layer shows a nearly linear trend for the lower values, which tends to plateau at higher alumina thickness (Figure S2). This can be explained by an efficient conversion of the metal oxide to Al-PMOF for thin alumina layers near the surface; when the alumina content increases, the growth of thicker crystals is observed, and after reaching a critical size, free-standing MOF crystals can be released into the solution, somehow limiting the thickness achievable on the surface. This analysis shows that ALD of the parent oxide allows a fine control of the Al-PMOF layer thickness on a sub-micrometer scale. However, it is important to note that lowering down the number of ALD cycles can induce defects in the metal oxide thin film, thus hindering the synthesis of homogeneous MOF thin films of much thinner (below 100 nm) thicknesses. 

### 2.3. Optical Properties

**Figure 5:**
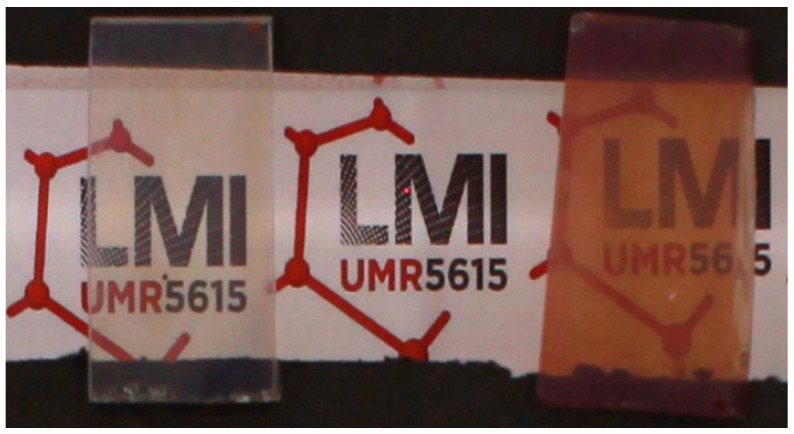
Photographs of MOF thin films in a controlled light environment for Al-PMOF from 200 cycles alumina ALD film (left) and Zn-Al-PMOF from 300 cycles alumina ALD film (right). To demonstrate the good transparency and clarity, the films were lifted 3 cm away from the surface.

**Figure 6:**
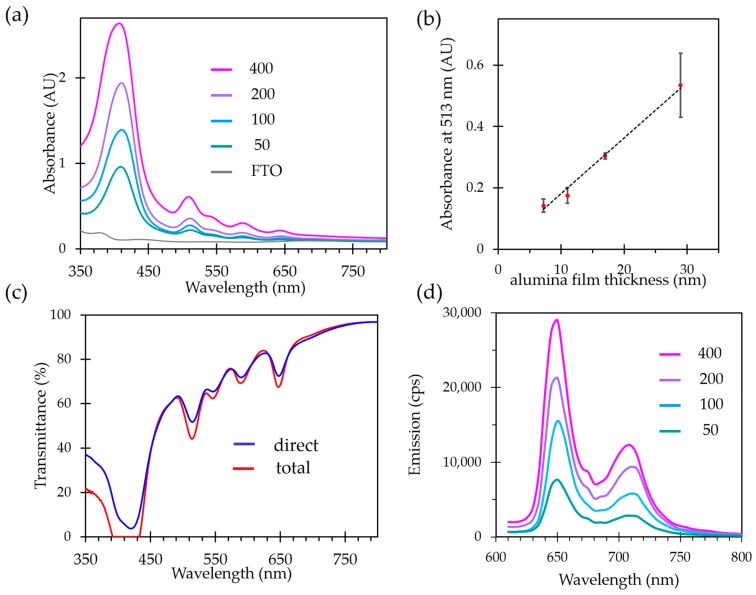
Optical properties of Al-PMOF thin films: UV-Vis absorbance for thin films of variable thicknesses (**a**), plot of the absorbance at 513 nm against the thickness of the parent alumina layer, with the standard deviations at each point (**b**), total and direct transmittance measured for Al-PMOF thin film grown from alumina layers deposited with 200 ALD cycles (**c**), emission spectra of Al-PMOF thin films of variable thickness at room temperature, using an excitation wavelength 418 nm (**d**).

The optical properties were investigated for the thin films grown on FTO substrates. Transparency of the film is an important feature for photoactive materials as it allows deep light penetration and reduces the light scattering. Classic thin films preparation methods such as spin or dip coating usually produce films with considerable opacity due to the surface roughness and lack of homogeneity, when layer-by-layer film growth can lead to higher optical quality films but with a very time-consuming process. Transparency and clarity of the films were first estimated by naked eye observations. As the distance between the object and the film has a strong influence on the clarity, the films were elevated 3 cm above the pattern. As evidenced by the photographs, a clear observation of pattern behind the sample is achieved (Figure 5). To evaluate quantitatively the thin films’ transparency, UV-vis absorption experiments were performed both in direct transmission geometry and using an integrating sphere to evaluate the part of the diffuse transmittance originating from the light scattering by the film. Both signals are plotted in Figure 6a. Light absorption characteristic of the porphyrin is observed in the two spectra. The transmittance values remain very close in the whole UV-vis range, attesting that a very little portion of the light is scattered by the film. 

Next, the UV-Vis absorption and emission properties were evaluated as a function of the film thickness, controlled by the number of ALD cycles used for the parent alumina growth. The absorption spectra of all the samples are characteristic of the free-base porphyrin ligand in Al-PMOF; the intensity of absorption bands increases with an increasing film thickness (Figure 6a). Measurements on a set of three samples prepared for each number of ALD cycles reveal a trend with the deviation values, demonstrating a good linearity between the thickness of the starting alumina layer and the absorption of the resulting Al-PMOF thin film (Figure 6b). Therefore, the absorption properties of the MOF film can be directly tuned by the number of ALD cycles. 

Free-base porphyrins are well known for presenting luminescence emission in the red-visible region; this feature is preserved in the bulk Al-PMOF as previously reported [13]. The emission spectra of Al-PMOF thin films were measured upon an excitation at 418 nm (Figure 6d). They display two signals in the red region, centered at 650 and 710 nm, in agreement with the transitions commonly reported for porphyrins in solution [20,21]. Figure 6d shows that the intensity of the emission increases linearly with the film thickness. It indicates that the light collection efficiency of the film remains unaltered as the film thickness increases, and that no quenching occurs with increasing number of photo-emissive molecules on the surface. This is allowed by the regular porphyrin structuring inside the MOF, as luminescence quenching due to the aggregation of emissive centers is usually observed for porphyrinic compounds without strong structuring [22,23,24] and in solution [21,25]. 

### 2.4. Accessible Porosity and Post Modification

Assessing the intrinsic porosity of thin films is challenging due to the very low total amount of material deposited. For this reason, classical volumetric analysis such as nitrogen sorption isotherms measurements at 77K are not applicable whereas ellipsometric porosimetry is well suited. This method relies on the monitoring of the optical properties’ change upon adsorption and desorption of a vapor. MOF films were first activated by heating at 120 °C to remove residual water from the pores. Note that the thermal stability of the thin films was checked, and the PXRD patterns (Figure S5) indicate that the film remains crystalline without any noticeable change after 4 h at 120 °C under air. After this activation step, the ellipsoporosimetry measurements were carried out at room temperature using water as probe molecule. Si-supported films were analyzed for ease of characterization. Figure S3 shows the plot of the refractive index variation upon adsorption and desorption of water vapor at room temperature. From these data, a pore radius of 0.5 nm and a surface area of ca. 850 m^2^·cm^−3^ were determined, in good agreement with Al-PMOF structure in terms of pore size and slightly below the reported BET surface area of the bulk sample (data deduced from the crystal structure for comparison: the material density of 0.81, BET surface area of 1400 m^2^·cm^−1^ corresponding to the volumetric surface area of 1134 m^2^·cm^−3^ and the larger pores of approx. 1 nm diameter). The slightly reduced BET SA can be due to the presence of a larger portion of defects in the film compared to the bulk sample.

**Figure 7:**
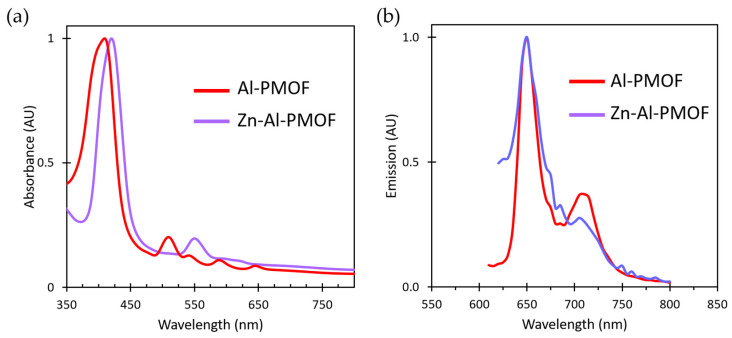
Evolution of UV-vis (**a**) and emission (**b**) spectra of the MOF thin film upon Zn insertion by VPI.

After evidencing the accessible porosity of the film, post-synthesis modification using vapor-phase infiltration (VPI) was attempted to chemically modify the framework and its photophysical properties. Derived from ALD, VPI method is based on the solid–vapor heterogeneous reactions that can proceed inside porous matter upon successive exposure to a vapor phase reagent. It is a well-suited method to perform chemical reactions inside porous materials [26]. Recently, we demonstrated that VPI is a fast and efficient way to modify bulk microporous MOFs [27]. Here, the films were subjected to diethylzinc (DEZ) exposures at 110 °C. Upon VPI, Zn(II) is chelated by the tetrapyrrolic core of the porphyrins which impacts the UV-vis absorption properties of the film. The VPI process was monitored by UV-vis spectroscopy and successful Zn incorporation inside Al-PMOF is demonstrated by the modification of the absorption and emission properties (Figure 7a,b). The Soret band is batochromically shifted from 418 to 425 nm and the Q absorption bands are reduced for Zn-Al-PMOF to two main bands at 550 and 605 nm. Regarding the luminescence properties, the film remains emissive with an intense fluorescence signal centered at 650 nm (Figure 7b). This emission energy is consistent with the reported data for Zn-metallated porphyrins [28,29]. In addition to offering a degree of tuning, the success of Zn VPI also proves the accessible porosity of the Al-PMOF thin films.

### 2.5. Photoelectrochemical Characterizations

Our methodology allows growing high optical quality tunable porphyrinic MOF thin films of controlled thickness on an electronic conducting substrate. As a proof of concept, photocurrent measurements were carried out on the film obtained starting from alumina deposited with 200 ALD cycles, thus corresponding to an MOF film thickness of ca. 420 nm.

**Figure 8:**
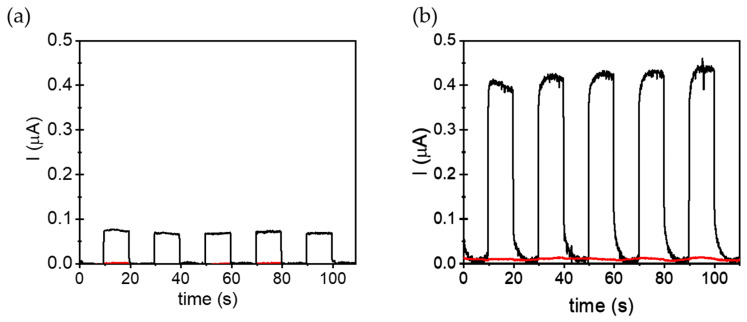
Chronoamperometry diagram showing photocurrent produced by Al-PMOF (black line), alumina (red line) thin films supported on FTO as WE polarized at +0.4 V and using Ar-purged acetonitrile solutions of TBAPF_6_ (0.2 M) in the absence (**a**) or in the presence of MeOH (**b**); every 10 s the samples are illuminated by simulated sunlight for 10 s.

The photoelectrochemical (PEC) characterizations were performed in a standard three-electrode cell system using a tetrabutylammonium hexafluorophosphate (TBAPF_6_) acetonitrile solution (0.1 M) as electrolyte. Al-PMOFs and ALD-grown alumina thin films supported on FTO were used as working electrodes (WE). The current density measurements of the positively polarized (+0.4 V) WEs were carried out under five consecutive on/off illumination cycles. Figure 8 shows that Al-PMOF WEs exhibit outstanding photocurrent densities compared to alumina ones. These measurements agree with the role of porphyrin-based units in PMOFs as active moieties to increase the photoinduced charge separation efficiency of FTO films coated with Al-PMOFs under simulated sunlight irradiation. To check the recyclability of the method, additional chronoamperometry experiments were performed up to forty consecutive on/off irradiation cycles to evaluate the stability and recyclability of the system. The results shown in Figure S4 evidence a small gradual photocurrent fatigue of about 15% compared to the initial values. Analogous experiments carried out in the presence of methanol in the electrolyte solution greatly increase the current density (Figure 8b). These results agree with the role of methanol acting as an electron donor. In this case, methanol is oxidized by the photogenerated holes present in the Al-PMOF thin film and, thus, decreases the charge recombination and increases the number of electrons giving the current intensity.

## 3. Materials and Methods

All chemicals were purchased from commercial sources and used without any further purification. The TCPP was purchased from Porphychem^®^ and FTO covered glass slides were obtained from Solems^®^ (square resistance 7 ohms).

### 3.1. Characterizations

X-ray diffraction of thin films was performed on a PANalytical XpertPro MRD diffractometer with a Cu Kα1 radiation (λ = 1.540598 Å) used with 40 kV and 30 mA settings in θ/θ mode, reflection geometry.

Scanning Electron Microscopy images were recorded on FEI Quanta 250 FEG and Zeiss Merlin Compact microscopes in the microscopy center of Lyon 1 University.

The thickness of Al_2_O_3_ films was verified by spectroscopic ellipsometry at an incident angle of 75° using a Semilab (SE-2000) ellipsometer equipped with ellipsoporosimetry (EPA) cell. Ellipsoporosimetry was performed to evaluate the porosity of the Al-PMOF films on Si. Measurements were thus carried out using the EPA cell in which variation of the relative humidity (RH) was induced: from 0% to 100% during the adsorption test and from 100% to 0% during the desorption of water. Prior measurement calibration of RH was realized. Spectroscopic ellipsometry measurements were then recorded at an incident angle of 75° in increments of 10% RH. Each acquisition lasted 1000 s. Data fit was realized with the “SAM suite” software version 1.7.6 using a Cauchy dispersion law. In the case of Al-PMOF films, the fitting range was reduced in order to exclude the absorption phenomena related to the porphyrin.

The surface roughness and topography were characterized by AFM using a commercial CSI-Nano Observer microscope operating in tapping mode with an “ACT” tip, which has a resonant frequency around 300 kHz.

Transmission UV-vis spectra were recorded using an SAFAS Monaco UV-mc2 spectrophotometer. Total transmittance UV-Vis measurements were performed using a PerkinElmer UV/VIS Lambda 365 spectrophotometer equipped with an integration sphere.

Photoluminescence measurements were carried out with the FS5 Spectrofluorometer from Edinburgh Instruments and using the solid-state SC-10 sample holder at room temperature and under ambient atmosphere. Emission spectra were obtained using 5 nm step with a dwell time of 3 s per step.

Photocurrent measurements were carried out using a Gamry Instruments potentiostat (model Interface 5000E) together with a conventional three-electrode quartz cell system. A platinum wire was used as counter electrode and an Ag/AgCl electrode in saturated KCl solution as the reference. Al-PMOFs or alumina thin films supported on fluorine-doped tin oxide (FTO) were used as working electrodes. Tetrabutylammonium hexafluorophosphate (TBAPF6) acetonitrile solution (0.2 M) was used as electrolyte. In some experiments, methanol (0.1 mL) was added to the acetonitrile solution as hole scavenger. Prior to the measurements, the system was purged with Ar for 20 min to remove the oxygen present in the cell. The current density on the working electrode polarized at +0.4 V was recorded under both dark and illumination conditions. Specifically, simulated sunlight irradiations were performed using a Xe-Hg lamp (ref: 150 W, Hamamatsu ref. L8253; Hamamatsu spotlight source L9566-04 and light guide A10014-50–0110) equipped with an air mass filter, AM 1.5 global, glass only (model: 81094; Newport).

### 3.2. Thin Film Growth Procedures

#### 3.2.1. Alumina Growth by ALD

Alumina thin films were grown by atomic layer deposition (ALD) onto FTO covered glass slides and Si wafer with native oxide, simultaneously. The Si wafer served as reference for thickness measurement. Depositions took place at 150 °C in a home-made reactor, working in a continuous flow. Trimethylaluminum and milli-Q water were used as metal and oxygen source and introduced sequentially by pneumatic ALD valves from their reservoirs, kept at 25 °C. Under a total carrier gas flow of 200 sccm, the valve opening time and the subsequent purge length were set at 0.3 and 15 s, respectively, for both reactants. Considering a growth per cycle of 1 Å, the number of ALD cycles was adjusted to obtain the desired oxide thickness.

#### 3.2.2. Synthesis of Al-PMOF Thin Films

In a 40 mL vial, 30 mg (0.037 mmol) of TCPP was dissolved in 6 mL of DMF (5 min sonication). While stirring (>500 rpm), 6 mL of ultra-pure water was added and solution let to stir for 5 min. Alumina coated FTO slides or Si wafers were then dipped inside the TCPP solution and the reaction was heated at 150 °C for 20 h (3 h heating and cooling ramps were used). The recovered samples were rinsed 3 times with DMF and soaked for 12 h in DMF to remove unreacted TCPP. Then, the samples were washed 3 times with absolute ethanol followed by soaking in the ethanol for 12 h. The films were finally dried in oven at 100 °C for 2 h.

#### 3.2.3. Vapor Phase Infiltration (VPI) of Al-PMOF Thin Films

Al-PMOF films were dried for 1 h at 110 °C in the ALD reactor at 5 mbar to remove any adsorbed solvent or water molecules. With the same background pressure (5 mbar), 150 DEZ VPI cycles were carried out at 110 °C using 0.3 s pulse, 15 s purge. Al-PMOF film samples were held on two pieces of glass to allow a gap between the lower face and the sample holder, thus allowing gas diffusion on both faces.

## 4. Conclusions

In conclusion, Al-PMOF multifunctional thin film fabrication from ALD-coated alumina solid substrates was investigated. The morphology and roughness of the MOF films were characterized as a function of the parent alumina layer thicknesses. Sub-micrometer thickness thin films grown on FTO are of high optical quality in terms coverage, homogeneity and transparency. Moreover, the absorption and emission properties originating from the porphyrinic linkers are preserved and no luminescence quenching is observed for increasing film thickness. The tuning of the MOF film features, especially its thickness and crystallite size, by the number of ALD cycles performed for the growth of the parent oxide is evidenced. The intrinsic porosity of the films was demonstrated and employed for the VPI post-synthesis functionalization with diethylzinc in the vapor phase. The photo-electrochemical properties were assessed and the occurrence of photoinduced charge separation of Al-PMOFs thin film supported on FTO electrode was evidenced upon positive polarization by photocurrent measurements under simulated sunlight irradiation. Overall, this work clarifies the Al-PMOF growth process from the parent oxide and demonstrates the optical properties of the MOF film as well as provides a successful proof of concept for the materials’ photoactivity through the photocurrent generation.

## Data Availability

The data presented in this study are available on request from the corresponding authors.

## Supplementary Materials

The supporting information can be downloaded at: https://www.mdpi.com/article/10.3390/molecules28155876/s1. Figure S1. (a) 10 × 10 µm and (b) 5 × 5 µm AFM images of Al-PMOF thin film on Si wafer obtained from 800 cycle and 400 cycle-Al_2_O_3_ ALD films, respectively. RMS roughness of approxiamtly 50–55 nm is determined indepdantly of the thickness of the starting oxide film. The bare Si substrate present a RMS roughness of 0.3 nm. Figure S2: MOF thin films on Si thickness deduced from cross-section SEM data, as a function of the ALD number of cycles (a) and as a function of ALD grown layer thickness (b). Figure S3: plot of the refracative index variation throughout adsorption and desorption of water vapour. Figure S4: Chronoamperogram showing photocurrent produced by Al-PMOF (black line) thin film supported on FTO as WE polarized at +0.4 V and using Ar-purged acetonitrile solution of TBAPF_6_ (0.2 M) under forty consecutive on/off simulated sunlight irradiation cycles every 10 s. Figure S5: PXRD patterns of Al-PMOF thin films on FTO grown from alumina depsoited in 100 ALD cycles, before (bottom) and after (up) a thermal treatment at 120 °C for 4 h.

## Sample Availability

Samples of the compounds are available from the authors.
