# Supplementary material for: Porphyrin-Based MOF Thin Film on Transparent Conducting Oxide: Investigation of Growth, Porosity and Photoelectrochemical Properties
Source: Molecules. 2023 Aug 4;28(15):5876. doi: 10.3390/molecules28155876 (PMC10421013; doi:10.3390/molecules28155876)
Supplement: Supplementary file 1 [file molecules-28-05876-s001.zip › molecules-2516052-supplementary.pdf]

# Supporting Information

# Porphyrin-Based MOF Thin Film on Transparent Conducting Oxide: Investigation of Growth, Porosity and Photoelectrochemical Properties

**Ben Gikonyo [1], Fangbing Liu [1], Saly Hawila [2], Aude Demessence [2], Herme G. Baldovi [3], Sergio Navalón [3], Catherine Marichy [1,*] and Alexandra Fateeva [1,*]**

[1] Laboratoire des Multimatériaux et Interfaces, Université Lyon, Université Claude Bernard Lyon 1, UMR CNRS 5615, F-69622 Villeurbanne, France
[2] Université Lyon, Université Claude Bernard Lyon 1, Institut de Recherches sur la Catalyse et l'Environnement de Lyon (IRCELYON), UMR CNRS 5256, F-69626 Villeurbanne, France; aude.demessence@ircelyon.univ-lyon1.fr (A.D.)
[3] Departamento de Química, Universitat Politècnica de València, C/Camino de Vera, s/n, 46022 Valencia, Spain; hergarba@cam.upv.es (H.G.B.); sernaol@doctor.upv.es (S.N.)
* Correspondence: catherine.marichy@univ-lyon1.fr (C.M.); alexandra.fateeva@univ-lyon1.fr (A.F.)

# Table of contents


AFM data SI3

MOF thin film thickness SI4

Ellipsometry porosimetry measurement of the Al-PMOF thin film SI5

Recyclability photocurrent experiments SI6

Thermal stability of Al-PMOF thin film SI7

- AFM data

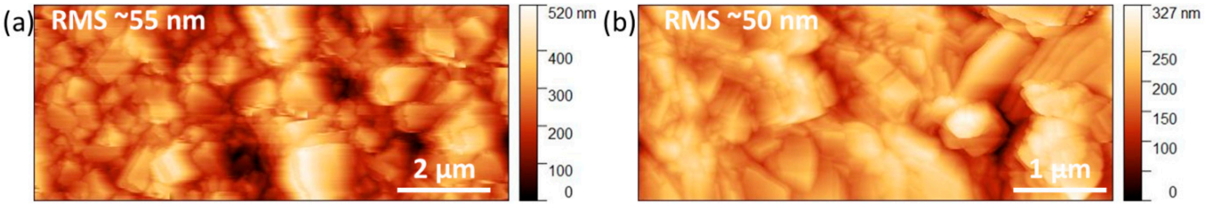


**Figure S1.** (**a**) 10x10 μm and (**b**) 5x5 μm AFM images of Al-PMOF thin film on Si wafer obtained from 800 cycle and 400 cycle-$Al_2O_3$ ALD films, respectively. RMS roughness of approxiamtly 50-55 nm is determined indepdantly of the thickness of the starting oxide film. The bare Si substrate present a RMS roughness of 0.3 nm.

♦ MOF thin films thicknesses

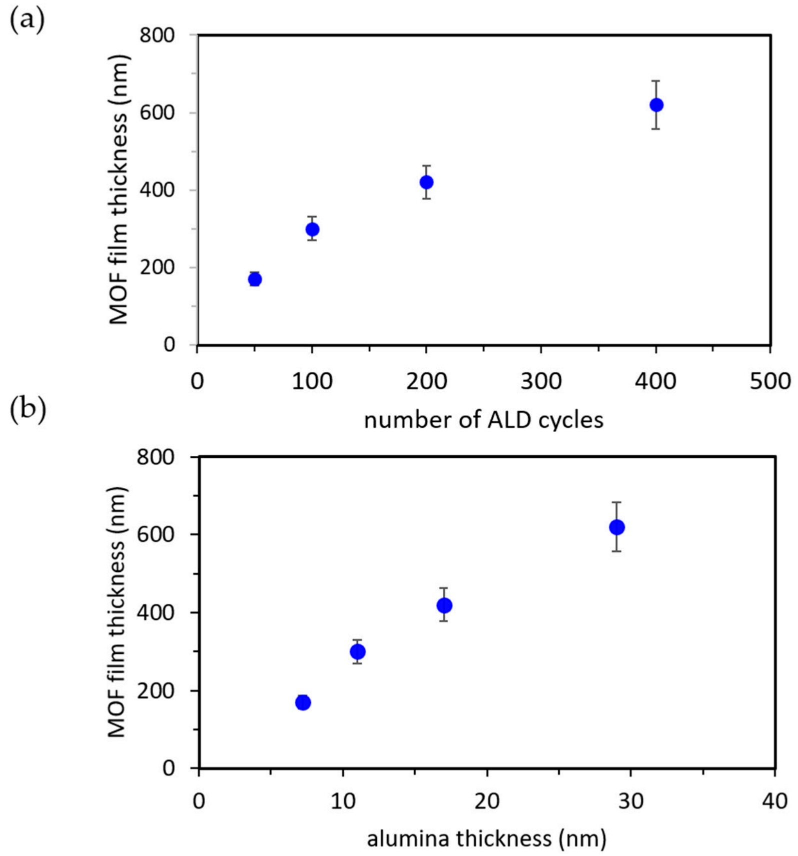


**Figure S2.** MOF thin films on Si thickness deduced from cross-section SEM data, as a function of the ALD number of cycles (**a**) and as a function of ALD grown layer thickness (**b**).

♦ Ellipsometry porosimetry measurement of the Al-PMOF thin film

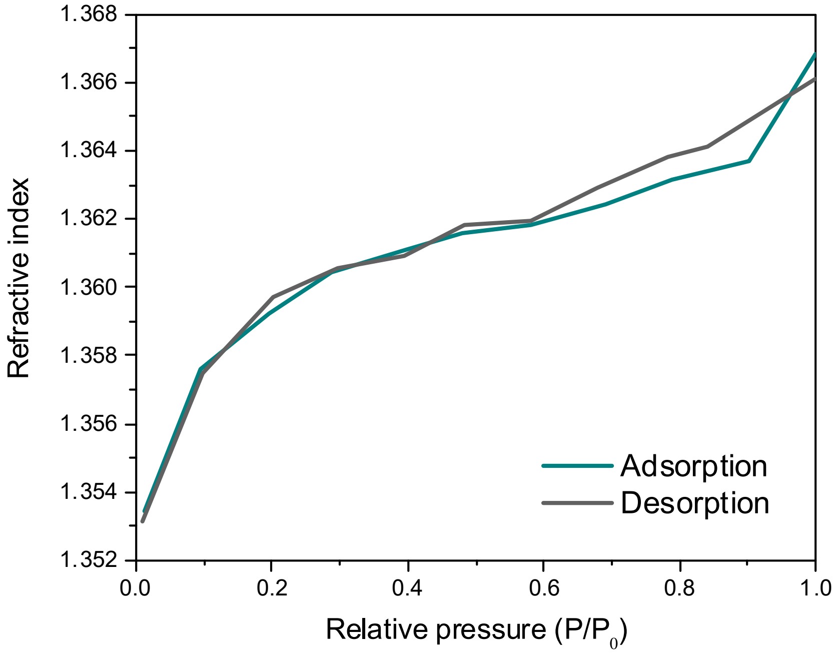


**Figure S3.** plot of the refracative index variation throughout adsorption and desorption of water vapour.

♦ Recyclability photocurrent experiments

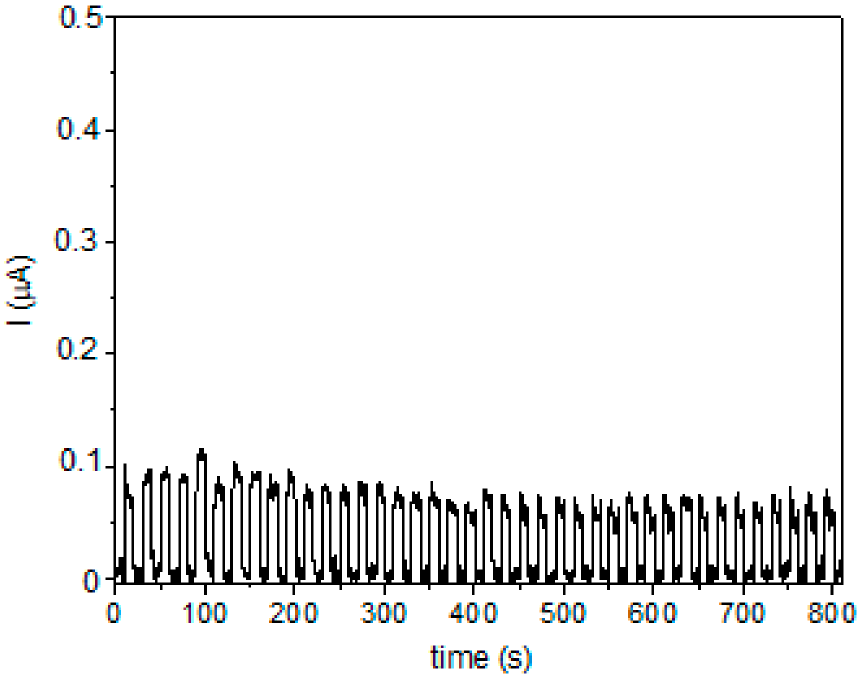


**Figure S4.** Chronoamperogram showing photocurrent produced by Al-PMOF (black line) thin film supported on FTO as WE polarized at +0.4 V and using Ar-purged acetonitrile solution of $TBAPF_6$ (0.2 M) under forty consecutive on/off simulated sunlight irradiation cycles every 10 s.

♦ Thermal stability of Al-PMOF thin film

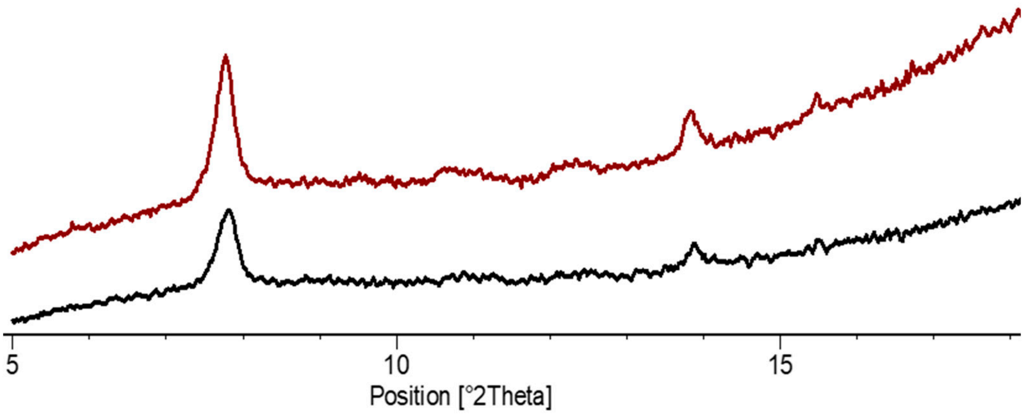


**Figure S5.** PXRD patterns of Al-PMOF thin films on FTO grown from alumina depsoited in 100 ALD cycles, before (bottom) and after (up) a thermal treatment at 120°C for 4 hours.
